# Obstruction of Ureter—Its Consequences and Remedy

**Published:** 1884-12-20

**Authors:** J. McF. Gaston

**Affiliations:** Professor of Principles and Practice of Surgery in the Southern Medical College, Atlanta, Georgia


### OBSTRUCTION OF URETER—ITS CONSEQUENCES AND REMEDY.

By J. McF. GASTON, M.D.,
Professor of the Principles and Practice of Surgery in the SOUTHERN MEDICAL COLLEGE, Atlanta, Georgia.

Having attended, in consultation with Prof. T. S. Powell, during the past month, a case of urinary infiltration from lesion of the ureter on the left side, in a female patient, it occurs to me that a report of the circumstances connected with its origin, progress, and termination may be appropriately accompanied with other facts growing out of troubles in this duct.

As was learned upon my first visit, the woman, about fifty years of age, had been in infirm health for a considerable time; and some days previously she had suffered from an attack of renal colic, which was followed by the protuberance which then existed in the iliac and inguinal regions. The fluctuation was very distinct in those regions, extending back even to the lumbar region, but confined to the space immediately within the crest of the ilium and Poupart's ligament, and, but for the history of the case, might have been mistaken for a psoas abscess. An incision with a bistoury was made into the most salient point, somewhat above a line running from the superior spinous process and the umbilicus, giving exit to a clear, straw-colored fluid, which was without perceptible odor, until at least a quart was evacuated, leaving the parts in a relaxed condition where the distention had existed.

A portion of the fluid discharged subsequently was analyzed by Prof. J. A. Burns, and found to be urine, as was supposed previously, from a rupture of the ureter by a renal calculus. No means were resorted to after the evacuation to maintain an external opening, and the cutaneous incision closed by first intention, so that, within three days, when the accumulation was again distending the tissues, it was requisite to use the bistoury again at the site of the former incision. At the close of the discharge on this occasion there was some pus mixed with the urine, and a drainage tube was retained in the opening, extending down towards the inguinal region, with a doubled strong cotton thread passed through one side of the extremity of the rubber tube and secured by a strip of cloth around the waist of the patient. The discharge was kept up continuously from this time ; and, after a few days, a solution of iodine, with iodide of potash, was injected into the cavity. As there was no further evidence of purulent discharges, it was apprehended that uremic poisoning had ensued, and it was hoped that this antiseptic application into the cellular tissue might arrest its progress ; but the decline of the vital forces progressed rapidly, and the patient sank on the 8th of November, being the fifteenth day from the original opening for the discharge of the infiltrated urine. No opportunity was given for a postmortem examination, and hence the exact location of the lesion of the ureter could not be determined ; but there can be no doubt as to the fact that this collection of urine outside of the peritoneum came from the ureter.

For the better understanding of the results of urinary infiltration from rupture of the ureter I may refer to two cases of abscess from this cause, under my observation, which were reported in the March number, for the present year, of Gaillard's Journal, as follows :

In this connection may be mentioned two more cases of abscess resulting from urinary obstruction in the ureters. One was in the person of a male, and the other of a female, subject. The former occurred in the practice of a medical friend, who gave me free access to the case, and the latter was under my personal care. In the early stages, each presented deep-seated fluctuations, which came gradually to the surface. Being explored, first with the trocar, and afterwards opened with the bistoury, there appeared in each case a discharge of pus with admixture of urine. In the male case there was a very considerable destruction of tissues, and the urinary abscess occupied a large space in the left iliac and hypogastric region, from which shreds of disorganized substances, mixed with pus and urine, were discharged for some weeks.

In the case of the female the cavity was not so extensive, and was confined to the hypogastric regions.

When this circumscribed abscess was first opened, about a quart of mingled pus and urine was discharged, and subsequently there was no considerable accumulation, as the aperture was tented, and the collection of pus and urine escaped constantly. Both of these abscesses were treated with injections of diluted phenic acid immediately after evacuating the contents, and for some time they were repeated daily ; but subsequently injections of a strong solution of the nitrate of silver were resorted to with salutary effects. The ravages caused by the urinary infiltration in the case of the man required some months for the restoration of the loss of substance, yet the process of reproduction was entirely satisfactory, and terminated in complete closure of the wound without even a fistulous opening. The extraordinary lesion of the ureter in this case was explained by the appearance of a small urinary calculus in the dressings, which had served as the cause of obstruction and ulceration of the ureter.

Nothing of this nature was discovered in the discharges from the abscess of the female, yet it might have escaped with the pus, when the abscess was opened, without attracting attention. Her case progressed most favorably throughout its course, and within one month there was simply a small discharge of urine from the wound, and, this ceasing gradually, the opening closed spontaneously without any further trouble.

The fortunate issue of these aggravated cases must be attributed, mainly, to the persistent use of the medicated injections, and I recommend this proceeding in similar cases, or in any instance of circumscribed abscess in the abdominal region. These antiseptic applications are not intended simply to cleanse the parts with which they come in contact, but to exert a therapeutic influence upon the tissues.

Though the notices of this accident are not very full or satisfactory, it may prove instructive to give a few quotations from standard authors.

Gross says : "Wounds and lacerations of the ureters are common, and cannot be distinguished as separate and independent lesions. The effects are usually fatal, the more common causes of death being peritonitis and diffuse absesses in the loin."

In Holmes' Surgery, Sir Henry Thompson states that "the ureter is liable to inflammation, which extends by it from the bladder to the kidney. It may be the subject either of stricture or of obliteration, which conditions are generally produced by injury caused by the passages or impaction of calculi ; occasionally from pressure by a tumor, rarely from external injury."

I am indebted to the kindness of my colleague, Dr. J. C. Olmsted, for the following extracts touching this subject :

### RUPTURE OF URETER.

Rupture of the ureter is a very rare injury. Mr. Soland has collected four cases, one of which recovered, after the evacuation by puncture, at intervals, of about two gallons of fluid resembling urine, the other cases terminating in death, during the first, fourth, and tenth weeks, respectively. In none of the cases does it appear that peritonitis was present, the urinary extravasation having occurred into the cellular tissue behind the peritoneum.—(*Vide Ashhurst's Surgery, page 366.*)

Stanley has related a remarkable case in which the ureter was ruptured by external violence, and in which the patient recovered; a very large accumulation of fluid forming on the injured side of the abdomen, with considerable circumscribed tumefaction and fluctuation, and which required frequent tapping. In another case, in which the pelvis of the kidney was ruptured, a similar collection of urine took place within the abdomen, requiring tapping ; as much as six pints being removed at one sitting. On examination, after death, which occurred in the tenth week from the accident, a large cyst was found behind the peritoneum, communicating with the pelvis of the kidney.—(*Vide Erichsen's Science and Art of Surgery, page 559.*)

The ureter sometimes ruptures as a consequence of injury from within, due especially to calculus, which either directly destroys the wall of the tube, or first originates ulceration, and this subsequently leads to perforation. This tube has also been cut across during the performance of operations, such as ovariatomy.—(*Vide Reynolds' System of Medicine, article by Fred. T. Roberts, page 742.*)

The rupture or perforation of the ureter is attended with grave consequences. The urine escapes, and sets up violent inflammation in the parts around, leading to the formation of abscesses, or to peritonitis, with the accompanying symptoms, both local and general. The TERMINATION IS ALWAYS FATAL.—(*Vide Roberts' article op. cit.*)

### TREATMENT.

Nothing can be done in cases of rupture of the ureter, except to keep the patient absolutely at rest, and to treat the consequences of the lesion. General treatment is indicated in certain forms of disease, similar to that described as applicable to the same affections when they involve the kidneys, of which they form a part.—(*Ibid.*)

CHARACTER OF HEALTHY URINE.

While still warm and fresh, the urine has a peculiar, but not offensive, odor.—( *Vide Dalton's Physiology.*)

While these cases of rupture of the ureter are amongst the gravest which the surgeon has to treat, Mr. Henry Morris, Surgeon to the Middlesex Hospital, London, claims the feasibility of removing from the ureter an impacted calculus when sufficiently near the vesical orifice to be felt with the finger.

In the American Journal of Medical Sciences, his method of operation is thus described : Having rapidly dilated the urethra, if the patient be a female, or opened the urethra in the median line, immediately in front of the prostate, if the patient be a male, the neck of the bladder should be passed by the index finger of the left hand, and a careful digital examination made of the bladder walls. If a hard, fixed body be felt, covered over by the bladder mucous membrane, at or near the orifice of one of the ureters, a gum lancet-shaped knife, on a long, slender shank, should be introduced along the left index finger, and with it an incision should be made through the tissue covering the calculus. The knife should then be carefully withdrawn, and a slender scoop or curette, introduced along the index finger of the left hand still retained within the bladder, should be employed for turning the calculus out of its bed. * * * It is further urged by Mr. Morris that, if no stone can be felt through the bladder, life may yet be saved by giving vent to the pent-up urine by lumbar nephrotomy.

It does not seem to have occurred to this English innovator that the catheterization of the ureter should be resorted to as a preliminary to this proceedure ; and, while this suggestion is not backed up by any record of practical results, the author may learn a lesson in conservative surgery from the following experience of an American surgeon, in the removal of renal calculi from the ureter by manipulation.

From the MEDICAL RECORD of November 22, 1884, it will be observed that Dr. J. P. Shafer, of Parkersburg, West Virginia, finding that a married lady, thirty-four years of age, was passing calculi through the ureter, concluded to help them along in the following manner : With his fingers upon the kidney, he pressed and pulled towards himself until he found that the seat of pain was changed to another point a little forward, and so continued this manipulation at intervals, removing, each time, the stone several inches, when he, as well as the patient, felt it pass from under his fingers into the bladder. So soon as the stone passed, the pain and spasms ceased. From this time forward, for the next ten days, she passed calculi every two days, the passage being assisted by Dr. Shafer. Then she passed no more for fourteen months, when the attacks began again, and continued in the same way, and with the same treatment. Dr. Shafer secured some of the calculi—one of which, he says, measured one to one and one-eighth inch in length by one-half to three-eighths inch in breadth. The patient is still living, and has been seen and manipulated by other physicians. The editor adds, that the woman is hysterical, indicating the probability of some voluntary or involuntary deception. But the only doubt that rests upon my mind is in regard to the good faith and responsibility of the operator, as the facts of the case are too clearly set forth to admit of any deception, except in regard to the trustworthiness of the observer ; and it is due to the history of such an operation that the names of his colleagues should be given in corroboration of the manipulations.

The location of the ureter, outside of the peritoneal sac, within the cellular tissue of the iliac region, so as to admit of a support to its walls in making such traction upon a solid body contained in the canal, renders the account plausible, and at least serves as encouragement in attempting this process of massage in similar cases. Only in the event of failure to effect relief in this mode would the alternative of Mr. Morris be warrantable, or cutting down upon the ureter be justifiable, for the removal of calculi.

